# Response to review article published titled ‘Total ankle arthroplasty versus ankle arthrodesis - a comparison of outcomes over the last decade’

**DOI:** 10.1186/s13018-019-1190-1

**Published:** 2019-05-20

**Authors:** Hasan R. Mohammad, Will Debrock, Stephen J. Mellon, Paul Cooke

**Affiliations:** 1 Nuffield Department of Orthopaedics, Rheumatology and Musculoskeletal Sciences, University of Oxford, Oxford, UK; 2 Nuffield Orthopaedic Centre, Oxford University Hospitals NHS Foundation Trust, Oxford, UK

## Abstract

We write in response to Lawton et al.’s (J Orthop Surg Res 12:76, 2017) important systematic review comparing the outcomes of total ankle replacement (TAR) and ankle arthrodesis (AA) after reviewing the existing literature. Traditionally, AA was the gold standard treatment for ankle osteoarthritis but there is renewed interest in TAR given modern design advantages of preserved ankle motion and gait. We outline some pertinent issues for surgeons to consider when interpreting results from review articles comparing treatment types given the limitations of primary studies. These include significant clinical heterogeneity from the indication for surgery, different treatment type subgroups and from poorly defined clinical outcomes.

Letter to the editor

We recently read with interest the article written by Lawton et al. [1] that was published in the Journal of Orthopaedic Surgery and Research and have conducted a literature search ourselves. This is an interesting review which addresses the pertinent question of whether ankle arthrodesis (AA) or total ankle arthroplasty (TAR) is the best treatment for end-stage ankle arthritis. Although traditionally AA was the gold standard, TAR offers significant advantages including ankle motion preservation and improved gait.

The above article highlights apparent and important limitations of current literature including a lack of direct comparative studies, cohorts with long-term follow-up and randomised controlled trials (RCTs). However, there are more subtle considerations which surgeons should consider when interpreting the clinical significance of pooled results from reviews comparing the outcomes of TAR and AA.

There is a great deal of clinical heterogeneity from primary studies investigating the outcomes of TAR and AA from multiple sources. From our own literature searches, the indication for surgery is rarely clearly defined and is often phrased as end-stage arthritis. This is a heterogenous definition which may include rheumatoid arthritis, neuropathic arthritis, traumatic osteoarthritis, and primary osteoarthritis. Each of these pathologies has specific but differing risks associated with surgery.

Furthermore, the surgical treatments used are often broad with no concise definition. TAR can be divided into subgroups by the implant’s generation and the number of components such as fixed (two-component systems) and mobile bearings TARs (three component systems) [2]. AA procedures are even more varied and can be performed by differing open methods or arthroscopically, and then fixed with internal (screws, rods, plates) or external fixation. Furthermore, primary studies are often unclear whether AA is limited to tibiotalar AA or whether this includes fusion of adjacent joints. It appears that Lawton et al. [1] understandably pooled data from all these procedure subgroups given primary study limitations.

Primary studies rarely define endpoints of interest including revision, reoperation, non-revision reoperation, and complications which may explain why there is no consensus over which treatment is superior. This is presumably why these endpoints were not clearly defined in Lawton et al.’s review [1] and really limits the validity of any pooled analysis conducted.

Additionally, the primary studies included in the analysis vary significantly in follow-up duration and the mean age of patients undergoing surgery. From Lawton et al.’s [1] review, Gross et al. [3] had 3.7 years follow-up compared to Wood et al.’s [4] 7.3 years. Some of the studies included in the pooled analysis do not even have a defined follow-up duration, clearly identifying primary study quality as an issue. There is also a difference in patients’ age at surgery in TAR and AA groups by about 8 years from Lawton’s et al.’s [1] review.

We have additionally identified other specific limitations to Lawton’s review. It remains unclear why an arbitrary cut off of 200 ankles for TAR and 80 ankles for AA was used as inclusion criteria. Perhaps this arbitrary cut-off was guided from a pilot search, but this potentially introduces a selection bias—with possible inclusion of poorer quality AA studies. It is also interesting that the overall sample size for TAR is 2239 ankles, compared to 608 ankles from AA. Furthermore, studies with lower numbers of ankles for AA were included. This is surprising and counterintuitive, given that AA is regarded as the gold standard, and has been performed more frequently and for longer.

We feel that given these points, there should have been a paragraph comprising a risk of bias summary for included studies, to make the quality of primary studies clear and that a summary of patient-reported outcome measures should have been included, given these are important to patients when consenting for surgery.

We agree with the authors’ conclusions that given a lack of high-quality direct comparative studies, the decision of which treatment option should be made on a case by case basis. However, we feel that no conclusions can be made from the pooled analysis, and there is little merit reporting this, given the limitations of primary studies in defining endpoints consistently, heterogeneous clinical indications for surgery, and imprecise surgical treatment definitions. Some clarity on which modality is superior for ankle osteoarthritis may be reached from the large multicentre TARVA (TAR versus AA) RCT currently underway [5]. There is a need for longer-term cohort studies investigating these treatments to allow for subgroup analysis for specific surgical treatments, population types, and indications for surgery. Additionally, any future work should clearly define study clinical endpoints and investigate patient-reported outcome measures.

## References

[1] Lawton CD, Butler BA, Dekker RG, Prescott A, Kadakia ARJJoos, Research. Total ankle arthroplasty versus ankle arthrodesis—a comparison of outcomes over the last decade. J Orthop Surg Res, 2017;12(1):76. 10.1186/s13018-017-0576-1 PMC5437567 28521779

[2] OrthopaedicsOne. Total ankle replacement (TAR). OrthopaedicsOne. Accessed [28/1/2019] 2012; Available from: https://www.orthopaedicsone.com/x/3oKdAQ.

[3] Gross CE, Lampley A, Green CL, DeOrio JK, Easley M, Adams S (2016). The effect of obesity on functional outcomes and complications in total ankle arthroplasty. Foot Ankle Int.

[4] Wood P, Prem H, Sutton CJTJob, volume jsB. Total ankle replacement: medium-term results in 200 Scandinavian total ankle replacements. J Bone Joint Surg Br, 2008;90(5):605-609. 10.1302/0301-620X.90B5.19677 18450626

[5] Goldberg AJ, Zaidi R, Thomson C, Doré CJ, Skene SS, Cro S (2016). Total ankle replacement versus arthrodesis (TARVA): protocol for a multicentre randomised controlled trial. BMJ Open.

